# Prospective, open, multi-centre phase I/II trial to assess safety and efficacy of neoadjuvant radiochemotherapy with docetaxel and oxaliplatin in patients with adenocarcinoma of the oesophagogastric junction 

**DOI:** 10.1186/1471-2407-13-75

**Published:** 2013-02-11

**Authors:** Markus Moehler, Ines Gockel, Hans-Peter Roessler, Dirk Arnold, Tanja Trarbach, Thomas Thomaidis, Gunther Klautke, Claus Rödel, Baruch Brenner, Hauke Lang, Peter R Galle, Carl C Schimanski, Heinz Schmidberger

**Affiliations:** 1 I. Medical Department, Johannes-Gutenberg University Mainz, Langenbeckstr.1, Mainz 55101, Germany; 2 IV. Medical Department, University Halle-Wittenberg, Halle, Germany; 3 West German Cancer Centre, University Hospital, Essen, Germany; 4 Radiotherapy Department, University Rostock, Rostock, Germany; 5 Radiotherapy Department, Johann-Wolfgang-Goethe University, Frankfurt, Germany; 6 Institute of Oncology, Davidoff Cancer Center, Beilinson Hospital, Rabin Medical Center, Petach-Tiqva, and Sackler Faculty of Medicine, Tel-Aviv University, Ramat Aviv, Tel-Aviv, Israel

**Keywords:** Docetaxel, Neoadjuvant radiochemotherapy, Chemoradiotherapy, Oesophagogastric cancer oxaliplatin

## Abstract

**Background:**

This phase I/II-trial assessed the dose-limiting toxicities (DLT) and maximum tolerated dose (MTD) of neoadjuvant radiochemotherapy (RCT) with docetaxel and oxaliplatin in patients with locally advanced adenocarcinoma of the oesophagogastric junction.

**Methods:**

Patients received neoadjuvant radiotherapy (50.4 Gy) together with weekly docetaxel (20 mg/m^2^ at dose level (DL) 1 and 2, 25 mg/m^2^ at DL 3) and oxaliplatin (40 mg/m^2^ at DL 1, 50 mg/m^2^ at DL 2 and 3) over 5 weeks. The primary endpoint was the DLT and the MTD of the RCT regimen. Secondary endpoints included overall response rate (ORR) and progression-free survival (PFS).

**Results:**

A total of 24 patients were included. Four patients were treated at DL 1, 13 patients at DL 2 and 7 patients at DL 3. The MTD of the RCT was considered DL 2 with docetaxel 20 mg/m^2^ and oxaliplatin 50 mg/m^2^. Objective response (CR/PR) was observed in 32% (7/22) of patients. Eighteen patients (75%) underwent surgery after RCT. The median PFS for all patients (n = 24) was 6.5 months. The median overall survival for all patients (n = 24) was 16.3 months. Patients treated at DL 2 had a median overall survival of 29.5 months.

**Conclusion:**

Neoadjuvant RCT with docetaxel 20 mg/m^2^ and oxaliplatin 50 mg/m^2^ was effective and showed a good toxicity profile. Future studies should consider the addition of targeted therapies to current neoadjuvant therapy regimens to further improve the outcome of patients with advanced cancer of the oesophagogastric junction.

**Trial Registration:**

NCT00374985

## Background

Carcinomas of the stomach and gastroesophageal junction are highly aggressive neoplasms and are the second most common cause of cancer-related deaths worldwide. During the past few decades, a shift in the localisation of the tumours has been observed.

While the incidence of distal gastric cancer has been decreasing, adenocarcinomas of the proximal stomach and the gastroesophageal junction, including Barrett’s carcinoma, has steadily risen. Approximately 50% of patients present with locally advanced disease at diagnosis and the prognosis of these patients remains poor with a five-year survival rate of less than 20% despite radical surgical R0 resection for curative intent [1].

Therefore, multimodal strategies have been introduced including different neoadjuvant RCT or perioperative chemotherapy to improve the outcome for these patients [2-5]. Compared to chemotherapy alone, which may decrease distant relapse rates, the primary objectives of neoadjuvant RCT are to downsize and downstage locally advanced tumours to increase R0 resection rates and to reduce local recurrence rates [6]. Furthermore, the excision of irradiated areas can result in lower long-term toxicity and early systemic therapy allows for better control of tumour micrometastases. Consequently, four randomised clinical trials investigated neoadjuvant RCT versus surgery alone in localised oesophageal cancer, including patients with tumours of the gastroesophageal junction [7-10]. Whereas some studies showed a significant survival advantage for neoadjuvant RCT [7,10,11], few other studies failed to do so [8,9]. In these trials, chemotherapy given in parallel to the radiotherapy was based on cisplatin with 5-fluorouracil (5-FU). However, combination regimens using newer agents may improve patient outcomes. The palliative therapy options for metastatic disease have clearly improved with oxaliplatin and docetaxel, showing fewer side effects than cisplatin and better clinical responses, respectively [12,13], but have not yet proven to be of tolerable and beneficial in the context of neoadjuvant chemoradiation. Thus, this binational multi-centre phase I/II trial examined a modern double regimen comprised of oxaliplatin and docetaxel combined with radiation therapy (RT).

## Methods

### Study population

Patients with histologically proven adenocarcinoma of the oesophagogastric junction (AEG II–III, according to the Siewert classification) with stage I–III disease (T3 N0–N3 to T4 N0–N3) and at least one measurable lesion according to RECIST criteria were eligible. Cancer of the oesophagogastric junction was defined according to the Siewert classification [14] whereas AEG type I is defined as adenocarcinoma of the distal oesophagus, AEG type II is defined as adenocarcinoma of the cardia and AEG type III is defined as subcardial adenocarcinoma with infiltration of the oesophagogastric junction. Only patients with AEG type II and type III were included into this study. Patients were required to be between 18 and 75 years of age with adequate organ function and a Karnofsky performance status of ≥ 70% at study entry. Patients with distant metastases and any previous palliative, adjuvant or neoadjuvant chemotherapy and/or radiotherapy or previous surgery of the primary tumour were excluded. Patients with any other tumour type (except basal skin cell carcinoma or *in situ* cervical carcinoma that had been successfully treated), symptomatic peripheral neuropathy as determined by the National Cancer Institute common toxicity criteria (NCI-CTC) grade ≥ 2, or other serious medical conditions, known hypersensitivity to platinum-based substances or pregnant or breast-feeding patients were also ineligible. Women of child-bearing potential were advised on contraception. All patients gave written informed consent before enrollment.

### Study aims and design

This was a non-randomised, multi-centre phase I/II trial conducted at six study sites in Germany and Israel (Registered trial NCT00374985 at clinical trials.gov). The design and conduct of the study complied with good clinical practice in accordance with the Declaration of Helsinki and all local requirements. Ethical approval was obtained for all participating institutions.

Patients received radiotherapy together with simultaneous chemotherapy consisting of docetaxel and oxaliplatin once weekly over 5 weeks. For treatment with docetaxel, pre-medication with dexamethasone was given and conventional supportive measures for nausea and vomiting were employed. Antiemetic prophylaxis included a 5-HT3 antagonist in combination with dexamethasone or methylprednisolone. Four to six weeks after completion of RCT, the patients were scheduled to undergo surgery.

The primary objective of the study was to assess the dose-limiting toxiticities (DLT) and the maximum tolerated dose (MTD) of the RCT based on the following three dose levels (DL) of chemotherapy:

DL 1: Docetaxel: 20 mg/m^2^ i.v. for 1 hour, oxaliplatin 40 mg/m^2^ i.v. for 2 hours on days 1, 8, 15, 22 and 29.

DL 2: Docetaxel: 20 mg/m^2^ i.v. for 1 hour, oxaliplatin 50 mg/m^2^ i.v. for 2 hours on days 1, 8, 15, 22 and 29.

DL 3: Docetaxel: 25 mg/m^2^ i.v. for 1 hour, oxaliplatin 50 mg/m^2^ i.v. for 2 hours on days 1, 8, 15, 22 and 29.

The standard 3 + 3 design was planned for dose escalation and 3 to 6 patients were enrolled in each cohort. Secondary objectives of the study comprised assessment of the response rate, resectability, progression-free survival and overall survival as well as toxicity of the RCT regimen. Protocol-specified standard dose delays and/or dose reductions were implemented based on toxicities experienced during the RCT in individual patients. Dose-limiting toxicities (DLT) were defined as any non-haematological toxicity (excluding alopecia, nausea and vomiting) of grade ≥ 3, or any haematological toxicity as follows: neutrophils < 0.5 × 10^9^/l for a minimum of 7 days or temperature > 38.5°C, thrombocytes < 25 × 10^9^/l which occurred during the RCT. The maximum tolerated dose (MTD) was defined as the highest dose that resulted in DLT in fewer than 1 in 3 or 2 in 6 patients in a patient cohort.

Radiotherapy was difined in the protocol, was identical at all dose levels and started on day 1 of the chemotherapy. A single dose of 1.8 Gy was administered once a day and five times a week for five weeks on days 1–5, 8–12, 15–19, 22–26 and 29–33 for a total dose of 45 Gy. The sixth week of therapy included a boost of three extra radiotherapy fractions at 1.8 Gy (days 36–38) up to a maximum total dose of 50.4 Gy. Radiotherapy was administered using the three-dimensional planning technique; megavoltage photon energy ≥ 6 MV was used. Computerized imaging was used to define the primary tumor mass and involved lymph nodes (gross tumor volume, GTV). The recommended margins around the GTV were 2 cm radially and 5 cm superiorly and inferiorly. Distal para-esophageal and abdominal celiac lymph nodes were included in the clinical target volume up to a total dose of 45 Gy. The boost volume covered the GTV with a 2-cm margin to all directions. Radiation exposure to lungs, heart, spinal cord, kidney, and liver limited within predefined constraints (e.g. the mean lung dose should not exceed 20 Gy).

Clinical assessments to confirm eligibility were to be completed within seven days before starting therapy. Investigations to measure disease, such as endosonography, laparoscopy and computed tomography (CT) of thorax and abdomen, were required within 28 days before treatment start. Vital signs, physical examination, clinical chemistry and haematology were assessed weekly during RCT as well as prior to surgery.

### Efficacy and safety assessment

Tumour assessments were performed at baseline (within 28 days prior to treatment start), prior to surgery after completion of the RCT and every 3 months post surgery. Tumour assessments were evaluated according to the RECIST criteria version 1.0. Safety was assessed by recording adverse events (AE) during the treatment period, which were graded according to NCI-CTC version 3.0.

### Statistical analysis

Due to the study design, the sample size was dependent on the number of patients treated per dose level and also dependent on the number of dose levels. Dose escalation was only to be performed if no DLT was observed in 3 patients. If one DLT was observed, then 3 more patients were to be treated at that dose level. If a second patient experienced DLT, then the MTD was defined Therefore, a formal sample size calculation was not applicable for phase I testing. All statistical analyses were performed using the SAS software package V. 9.1.3 (SAS Institute, Cary; NC, USA) on the Windows platform.

Time-to-event data was analysed with the Kaplan-Meier method. Overall survival was defined as the time from the date of the start of RCT to death of any cause. Patients alive at their last follow-up were censored. The distribution of overall survival was estimated using the Kaplan-Meier method. Based on these curves, the estimated one and two-year survival rates were extrapolated together with their associated 95% confidence limits. Progression-free survival was analysed at the same time. Progression-free survival time was defined as the time from the start of chemotherapy to documented progression according to RECIST criteria or death, whichever occurred first.

## Results

### Patients

A total of 24 patients were enrolled at six participating institutions. Table 1 summarises the patient characteristics at baseline. The median age was 62.0 years (33–75 years) and there were 21 male (88%) and 3 female (13%) patients. All patients presented with a good Karnofsky performance status at study entry with a median of 95%, ranging from 90 to 100%. For the majority of patients (75%), the tumour was localised in the cardia (AEG II).

**Table 1:** Patient characteristics at baseline per dose level

**Characteristics**	**Dose level**			
	**1**	**2**	**3**	**Total**
Number of patients	4 (100%)	13 (100%)	7 (100%)	24 (100%)
**Age, years**				
N	4	13	6	23
Median (range)	55.5 (38–75)	65.0 (33–70)	56.0 (44–63)	62.0 (33–75)
**Gender, n (%)**				
Female	1 (25%)	2 (15%)		3 (13%)
Male	3 (75%)	11 (85%)	7 (100%)	21 (88%)
**KPS**				
N	3	12	7	22
Median (range)	100 (100–100)	90 (90–100)	100 (90–100)	95 (90–100)
**Siewert classification**				
AEG II	2 (50%)	9 (69%)	7 (100%)	18 (75%)
AEG III	2 (50%)	4 (31%)		6 (25%)
**T staging**				
3	3 (75%)	10 (77%)	6 (86%)	19 (79%)
4	1 (25%)	2 (15%)	1 (14%)	4 (17%)
X		1 (8%)		1 (4%)
**N staging**				
0		2 (15%)		2 (8%)
1	2 (50%)	5 (38%)	4 (57%)	11 (46%)
2	2 (50%)	4 (31%)	2 (29%)	8 (33%)
X		2 (15%)	1 (14%)	3 (13%)
**M staging**				
0	3 (75%)	12 (92%)	7 (100%)	22 (92%)
X	1 (25%)	1 (8%)		2 (8%)
**UICC staging**				
II A		2 (15%)		2 (8%)
III	4 (100%)	8 (62%)	6 (86%)	18 (75%)
NK		3 (23%)	1 (14%)	4 (17%)

KPS, Karnofsky Performance Status.

UICC staging according to the 6th edition: NK in case of unknown T or N staging (Tx, Nx).

### Safety and toxicity

Three patients were planned to be enrolled in dose level 1. Instead, four patients were recruited as two patients were enrolled at the same time. DLT occurred in one patient (grade 3 non-haematological toxicity: fatigue, dehydration and syncope). As this toxicity was considered to be unrelated to chemotherapy, the dose was escalated to the next level. At dose level 2, one DLT (grade 4 thrombocytopenia) was observed in the first cohort of three patients. Therefore, the cohort was expanded and another five patients were enrolled. As no further DLT occurred, the dose was escalated to dose level 3. Again, three patients were enrolled. Of these, one patient experienced heartburn/dyspepsia grade 4 and one patient developed fatigue grade 3. When expanding the cohort for another 4 patients, further DLT were observed (Fatigue, diarrhea, infection) (Table 2). Dose level 2 was therefore declared the MTD and an additional five patients were recruited at this dose level.

**Table 2:** Dose-limiting toxicities per dose level

	**Dose level**						
	**1**		**2**		**3**		**Total**
Number of patients	4 (100%)		13 (100%)		7 (100%)		24 (100%)
Any DLTs?							
No	3 (75%)		12 (92%)		2 (29%)		17 (71%)
Yes	1 (25%)		1 (8%)		5 (71%)		7 (29%)
If yes:							
CTC-Grade	III	IV	III	IV	III	IV	
Non-haematological toxicity grade ≥ 3:							6 (25%)
- Dehydration, syncope	1						
- Heartburn/dyspepsia						1	
- Fatigue					2		
- Diarrhea					1		
- Infection					1		
Haematological toxicity:							1 (4%)
- Thrombocytpenia				1			

DLT, dose limiting toxicity.

Table 3 summarises the frequency distribution of all adverse events of at least CTC grade 3. The most frequently observed grade ≥ 3 adverse events were nausea (25.0%), vomiting (20.8%) and fatigue (20.8%). Severe sensory neuropathy did not occur and haematological toxicity was infrequent and was only observed in three patients.

**Table 3:** Frequency distribution of all drug-related adverse events of at least grade 3

	**Dose level**			
**Event**	**1**	**2**	**3**	**Total**
Number of patients	4 (100%)	13 (100%)	7 (100%)	24 (100%)
Nausea	1 (25%)	2 (15%)	3 (43%)	6 (25%)
Vomiting	2 (50%)	2 (15%)	1 (14%)	5 (21%)
Dehydration			1 (14%)	1 (4%)
Diarrhoea			1 (14%)	1 (4%)
Dysphagia		1 (8%)		1 (4%)
Heartburn/dyspepsia			1 (14%)	1 (4%)
Fatigue	1 (25%)	1 (8%)	3 (43%)	5 (21%)
Pain			1 (14%)	1 (4%)
Platelets		1 (8%)	1 (14%)	2 (8%)
Leukocytes			1 (14%)	1 (4%)
Neutrophils			1 (14%)	1 (4%)

In eleven patients, chemoradiation was delayed once (Table 4). Adverse events were the reason for delay in five patients. Chemotherapy was delayed due to administrative reasons or due to patient / investigator decision in six patients. A total of 20 patients received full-dose chemotherapy and dose reductions due to adverse events were only required in three patients. Radiotherapy could be administered as planned total dose of 45 Gy followed by a boost of 3 times 1.8 Gy in 21/24 (88%) of all cases. One patient discontinued radiotherapy due to thrombopenia (final dose 39,6 Gy) and 2 patients discontinued due their patients wish (final doses 41,4 and 10,8 Gy). The total median dose was 45 Gy (range 10.8–50.4). Since the causal relationship of adverse events with treatment had been requested by a total yes-no, specific toxicity of radiotherapy was not recorded separately. However, no radiation-specific skin toxicities or or post operation complications were attributed to the part of radiation (Tables 3 and 5).

**Table 4:** Administration of radiochemotherapy

	**Dose level**			
	**1**	**2**	**3**	**Total**
Number of patients	4 (100%)	13 (100%)	7 (100%)	24 (100%)
Chemotherapy delay?				
No	3 (75%)	5 (38%)	5 (71%)	13 (54%)
Yes	1 (25%)	8 (62%)	2 (29%)	11 (46%)
If yes, reason for delay:				
Administrative reason	1 (100%)	4 (50%)	1 (50%)	6 (55%)
Adverse event		4 (50%)	1 (50%)	5 (45%)
Chemotherapy reduction?				
No	3 (75%)	11 (85%)	6 (86%)	20 (83%)
Yes	1 (25%)	2 (15%)	1 (14%)	4 (17%)
If so, reason for reduction:				
Administrative reason		1 (50%)		1 (25%)
Adverse event	1 (100%)	1 (50%)	1 (100%)	3 (75%)
Radiotherapy total dose (Gy)				
Mean (SD)	46.8 (4.41)	44.45 (10.68)	46.54 (2.63)	45.45 (8.07)
Median (range)	47.7 (41.4–50.4)	45.0 (10.8–50.4)	45.0 (45.0–50.4)	45.0 (10.8–50.4)

**Table 5:** Frequency distribution of all adverse events reported after surgery

	**Dose level**			
**Event**	**1**	**2**	**3**	**Total**
Number of patients	3 (100%)	11 (100%)	4 (100%)	18 (100%)
Any event	1 (33%)	2 (18%)	1 (25%)	4 (22%)
Pericardial effusion			1 (25%)	1 (6%)
Fever	1 (33%)			1 (6%)
Infection		1 (9%)		1 (6%)
Pleural effusion		1 (9%)		1 (6%)

Post-operative complications were reported in four patients and consisted of pericardial effusion, pleural effusion, fever or infection each in one patient (Table 5). One patient died due to pleural effusion and pneumothorax 9 days after surgery.

### Efficacy

Efficacy is summarised in Table 6. For 22 patients, overall response data prior to surgery was available. Objective response (CR/PR) according to the RECIST criteria was observed in seven patients (32%) in total. Two partial responses were observed at dose level 1 (two out of 3 patients), three patients achieved PR at dose level 2 (three out of twelve patients (25%)) and two patients achieved PR at dose level 3 (two out of seven patients (29%)).

**Table 6:** Surgical results and overall response

	**Dose level**			
	**1**	**2**	**3**	**Total**
Number of patients	4 (100%)	13 (100%)	7 (100%)	24 (100%)
Surgery?				
No	1 (25%)	2 (15%)	3 (43%)	6 (25%)
Yes	3 (75%)	11 (85%)	4 (57%)	18 (75%)
If so:				
R0	2 (67%)	8 (73%)	4 (100%)	14 (78%)
Grading				
G2		3 (27%)	1 (25%)	4 (22%)
G3	2 (67%)	4 (36%)	2 (50%)	8 (44%)
Information missing	1 (33%)	4 (36%)	1 (25%)	6 (33%)
Response prior to surgery (RECIST)				
N	3 (100%)	12 (100%)	7 (100%)	22 (100%)
PR	2 (67%)	3 (25%)	2 (29%)	7 (32%)
SD		5 (42%)	2 (29%)	7 (32%)
PD	1 (33%)	2 (17%)	2 (29%)	1 (5%)
NK/ND		2 (17%)	1 (14%)	2 (9%)
Staging prior to surgery (UICC)				
N	4 (100%)	12 (100%)	7 (100%)	23 (100%)
II A		4 (33%)	1 (14%)	5 (229%)
II B		1 (8%)	1 (14%)	2 (9%)
III	3 (75%)	4 (33%)	2 (29%)	9 (39%)
IV			1 (14%)	1 (4%)
NK/ND	1 (25%)	3 (25%)	2 (29%)	6 (26%)
Pathologic response [15]				
N	3 (100%)	11 (100%)	4 (100%)	18 (100%)
CR		4 (36%)	2 (50%)	6 (33%)
PR	1 (33%)	4 (36%)	2 (50%)	7 (39%)
SD		1 (9%)		1 (6%)
PD	1 (33%)	1 (9%)		2 (11%)
NK/ND	1 (33%)	1 (9%)		2 (11%)
Staging after surgery (UICC)				
N	3 (100%)	11 (100%)	4 (100%)	18 (100%)
0		2 (18%)	1 (25%)	3 (17%)
I		1 (9%)	1 (25%)	2 (11%)
II A	1 (33%)	2 (18%)		3 (17%)
II B	1 (33%)	2 (18%)	2 (50%)	5 (28%)
III		1 (9%)		1 (6%)
NK/ND	1 (33%)	3 (27%)		4 (22%)

A total of 18 patients underwent planned surgery (75%). Reasons for not undergoing surgery were insufficient performance status, concomitant medical conditions and patient request. R0 resection was documented for 14 patients. No information regarding R classification was available for four patients. Concordantly, a down-staging could be achieved in a substantial number of patients: at baseline, 18/24 (75%) patients presented with UICC stage III disease, whereas prior to surgery, only 10/24 (42%) patients had stage III disease by CT scan and/or endosonography. Pathologic response information [15] was available in 13/18 resected patients: 6 patients showed pCR, while 7 patients showed pPR. Despite intensified efforts, no detailed informations from pathology reports were available for the other five patients. Following surgery, there were remarkably only 2 patients with stage III disease according to the pathological reports.

**Figure 1:**
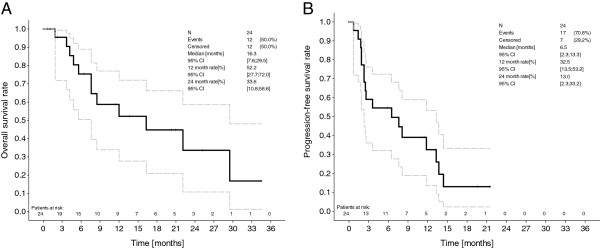
Kaplan Meier overall survival (A) and progression-free survival (B) in dose levels 1–3.

The median progression-free survival of the intent-to-treat-analysis for all patients (n = 24) was 6.5 months (Table 7, Figure 1). Median progression-free survival for patients at dose level 1 (n = 4) was 2.25 months, for patients at dose level 2 (n = 13) 6.5 months, and for patients at dose level 3 (n = 7) 13.5 months. The median overall survival for all patients (n = 24) was 16.3 months (95% CI [7.6; 29.5]). Median overall survival for patients at dose level 1 (n = 4) was 8.5 months (95% CI [5.6; 12.0]), for patients at dose level 2 (n = 13) 29.5 months (95% CI [4.8;x ]), and for patients at dose level 3 (n = 7) 22.1 months (95% CI [16.3; 22.1]). Regarding response rate and overall survival, it should be taken into consideration that not all patients underwent surgery (n = six patients (25%)), and in these the treatment was definitive RCT rather than neoadjuvant therapy.

**Table 7:** Progression-free survival (PFS) and overall survival (OS)

	**Dose level**			
	**1**	**2**	**3**	**Total**
Number of patients	4 (100%)	13 (100%)	7 (100%)	24 (100%)
PFS (months)				
Median (95% CI)	2.25 (1.4, 8.1)	6.5 (2.5, n.a.)	13.5 (2.3, 14.4)	6.5 (2.3, 13.3)
OS (months)				
Median (95% CI)	8.5 (5.6, 12.0)	29.5 (4.8, n.a.)	22.1 (16.3, 22.1)	16.3 (7.6, 29.5)

## Discussion

In recent years, perioperative strategies and neoadjuvant protocols have achieved significant advantages in terms of overall survival in patients with advanced oesophagogastric cancer. With the exception of one randomised trial [6], the proportion of individual localisations remains unclear, because all other trials enrolled patients with adenocarcinoma of the oesophagus, of the oesophagogastric junction and of the stomach. Hence this trial was designed to specifically address the issue of gastroesophageal tumours. In the treatment of gastroesophageal tumours, neoadjuvant RCT can achieve tumour shrinkage, leading to an increase of R_0_ resection rates, which in turn increases time to overall survival or even chance for cure. At the time this trial was designed, little data were available that assessed oxaliplatin and docetaxel with regards of safety and efficacy. Therefore, the aim of our study was to investigate the safety and tolerability of perioperative RCT with docetaxel and oxaliplatin in patients with tumours of the oesophagogastric junction.

Overall, the treatment regimen using oxaliplatin and docetaxel in combination with radiotherapy was well tolerated, with most frequently observed ≥ CTC grade 3 drug related adverse events being nausea (25%), vomiting (21%) and fatigue (21%). Hematological toxicities of > CTC grade 3 were observed to a lesser extent with thrombopenia in 8% of patients and leukopenia in 4% of patients.

Chemotherapy for GEJ tumours as part of combined RCT was previously based on cisplatin and 5-fluorouracil. Using CF as a neoadjuvant treatment (for 2 cycles prior to surgery and for a total of 6 cycles in case of good tolerability), neutropenia (20.2%) occurred in more than 10% of patients in a trial by Ychou et al. [16]. In another trial, using CF as sole treatment, neutropenia (57%), stomatitis (27%), and lethargy (14%) were reported for a substantial number of patients [17]. A study by Cunningham [12] has shown that replacing cisplatin for oxaliplatin for treatment of advanced gastric cancer can significantly lower the incidence of toxic side effects. Even it is not possible to directly compare chemotherapy alone with its combination with radiation, docetaxel plus oxaliplatin reported less toxicities and less proportions of deaths due to adverse events than its triple combination with capacitabine in a recent randomized phase II trial [18]. Despite its inferiority in efficacy, such a double protocol may thus be a better combination partner in RCT. Comparably, the RCT combination of paclitaxel and carboplatin has recently been studied in the CROSS-trial [19] randomizing 363 patients to either neoadjuvant radiochemotherapy followed by surgery, or surgery alone. Here, even if esophageal cancers were included mainly, gastroesophagel junctions tumors were also addressed. Toxicities in this study were also low, with hematologic toxicities occurring in 7% of patients and non-hematologic toxicities in less than 5% of patients.

Overall, combinations of taxanes and/or oxaliplatin with concurrent radiotherapy have been investigated only in a limited number of investigations [20-22]. Spigel et al [22] reported nausea and vomiting in 16%, and fatigue in 12% of patients treated with oxaliplatin, docetaxel and capecitabine. They have also found a significant amount of other drug related ≥ CTC grade 3 toxicities (anorexia, dehydration, esophagitis, and pulmonary symptoms). Solomon et al. [23] also reported fatigue (25%) and diarrhea (31%) to be the most frequently observed events, along with nausea (6.25%). These toxicity data are comparable with our observations and show that RCT with oxaliplatin and docetaxel can be safely administered with a dose of 20 mg/m^2^ docetaxel and 50 mg/m^2^ oxaliplatin as in DL2, but higher doses of docetaxel lead to more haematological and non-hematological toxicities. Comparably, doses of onother preoperative RCT phaseI/II study corresponded to our DL1 for docetaxel and oxaliplatin, however with a lower radiation dose but additionally combined with capecitabine [22].

In our trial, the progression-free survival of 6.5 months was somewhat lower than expected from other trials [22][24,25], while the 16.3 months overall survival compared to the rates reported by others [7,8], but was significantly lower than reported for the CROSS trial, where a median overall survival of 49 months in the RCT arm and 26 months in the surgery alone arm was achieved [19]. It should be noted that no final conclusions can be drawn with respect to treatment efficacy out of our study with a relatively low number of patients and wide confidence intervals. Again, 25% of the patients did not undergo surgery for various reasons (insufficient performance status, concomitant medical conditions and patient request). Even though this rate is substantially higher than the rate observed in the CROSS trial, where only 10% of neoadjuvant RCT treated patients could not be resected, it is comparable to those achieved in other trials [19,22].

## Conclusion

In summary, from the data presented, we conclude that docetaxel/oxaliplatin in combination with radiation is a safe neoadjuvant strategy for treatment of gastroesophageal junction cancers. Efficacy endpoints should be confirmed in larger clinical trials, preferably including newer biological agents and again preferably focusing on specific esophagogastric sites to omit different possible post-operative morbidity and mortality rates [26,27].

## Competing interests

M. Moehler D. Arnold, T. Trarbach and C. Schimanski have reported honoraria or a consultancy role for Sanofi-Aventis during the trial. The University of Mainz has received funding for Moehler’s study from Sanofi-Aventis, Germany. All other authors have declared no further potential conflicts of interest.

## Authors’ contributions

MM, IG, BB, CCS, and HS participated in the design of the study and performed the statistical analysis. MM, PR, DA, TTh coordinated and helped to draft the manuscript. All authors participated actively in the patient recruitment, read and approved the final manuscript.

## Pre-publication history

The pre-publication history for this paper can be accessed here:

http://www.biomedcentral.com/1471-2407/13/75/prepub
